# Identification of *Rtl1*, a Retrotransposon-Derived Imprinted Gene, as a Novel Driver of Hepatocarcinogenesis

**DOI:** 10.1371/journal.pgen.1003441

**Published:** 2013-04-04

**Authors:** Jesse D. Riordan, Vincent W. Keng, Barbara R. Tschida, Todd E. Scheetz, Jason B. Bell, Kelly M. Podetz-Pedersen, Catherine D. Moser, Neal G. Copeland, Nancy A. Jenkins, Lewis R. Roberts, David A. Largaespada, Adam J. Dupuy

**Affiliations:** 1 Department of Anatomy and Cell Biology, Roy J. and Lucille A. Carver College of Medicine, University of Iowa, Iowa City, Iowa, United States of America; 2 Masonic Cancer Center, Department of Genetics, Cell Biology and Development and Center for Genome Engineering, University of Minnesota, Minneapolis, Minnesota, United States of America; 3 Center for Bioinformatics and Computational Biology, Department of Opthalmology and Visual Sciences, Roy J. and Lucille A. Carver College of Medicine, University of Iowa, Iowa City, Iowa, United States of America; 4 Department of Genetics, Cell Biology and Development and Center for Genome Engineering, University of Minnesota, Minneapolis, Minnesota, United States of America; 5 Division of Gastroenterology and Hepatology, College of Medicine, Mayo Clinic and Mayo Clinic Cancer Center, Rochester, Minnesota, United States of America; 6 Cancer Research Program, The Methodist Hospital Research Institute, Houston, Texas, United States of America; National Cancer Institute, United States of America

## Abstract

We previously utilized a Sleeping Beauty (SB) transposon mutagenesis screen to discover novel drivers of HCC. This approach identified recurrent mutations within the *Dlk1-Dio3* imprinted domain, indicating that alteration of one or more elements within the domain provides a selective advantage to cells during the process of hepatocarcinogenesis. For the current study, we performed transcriptome and small RNA sequencing to profile gene expression in SB–induced HCCs in an attempt to clarify the genetic element(s) contributing to tumorigenesis. We identified strong induction of *Retrotransposon-like 1* (*Rtl1*) expression as the only consistent alteration detected in all SB–induced tumors with *Dlk1-Dio3* integrations, suggesting that *Rtl1* activation serves as a driver of HCC. While previous studies have identified correlations between disrupted expression of multiple *Dlk1-Dio3* domain members and HCC, we show here that direct modulation of a single domain member, *Rtl1*, can promote hepatocarcinogenesis *in vivo*. Overexpression of *Rtl1* in the livers of adult mice using a hydrodynamic gene delivery technique resulted in highly penetrant (86%) tumor formation. Additionally, we detected overexpression of *RTL1* in 30% of analyzed human HCC samples, indicating the potential relevance of this locus as a therapeutic target for patients. The *Rtl1* locus is evolutionarily derived from the domestication of a retrotransposon. In addition to identifying *Rtl1* as a novel driver of HCC, our study represents one of the first direct *in vivo* demonstrations of a role for such a co-opted genetic element in promoting carcinogenesis.

## Author Summary

HCC is the third deadliest cancer worldwide, largely due to a lack of effective treatment options. Therapeutic approaches targeted at the molecular mechanisms underlying tumor formation and progression have shown great efficacy for treating other tumor types. Unfortunately, however, much remains to be learned about the molecular pathogenesis of HCC. There is an urgent need to identify and characterize genetic alterations that drive HCC in order to facilitate the development of more effective targeted therapeutics for patients. Here, we present data showing that recurrent mutations identified in a mouse model of HCC result in overexpression of the *Rtl1* gene. We have validated *Rtl1* as a driver of HCC by demonstrating that its overexpression in mouse liver causes tumor formation. We also detected overexpression of this gene in a significant proportion of human HCC samples, suggesting that it may be a relevant therapeutic target for patients with this disease.

## Introduction

Hepatocellular carcinoma (HCC) is the third leading cause of cancer-related deaths worldwide [1]. In contrast to the downward trends in incidence observed for most cancer types, that of HCC continues to rise, particularly in the United States [2]. This is due in part to increases in obesity and hepatitis C viral infection, both of which have been implicated in HCC pathogenesis. Treatment options for patients are limited, particularly for those with advanced disease, and the five-year survival rate remains low at ∼10%.

A major goal of HCC research is to develop therapies targeted at the molecular mechanisms underlying tumor development and progression. This type of approach is expected to be much more efficacious, increasing survival rates for HCC patients. Consistent with this idea, treatment with sorafenib, a multi-kinase inhibitor, has shown survival benefits for late-stage patients [3] – a rare achievement in HCC treatment. Nevertheless, sorafenib treatment is only able to extend median survival by three months, underlying the need for improved targeted therapies. Unfortunately, the molecular drivers of HCC remain poorly characterized, precluding the development of such therapeutics. Large-scale sequencing efforts currently being undertaken by The Cancer Genome Atlas (TCGA) project will likely characterize the recurrent genetic alterations present in human liver tumors and may identify novel therapeutic targets. However, it is becoming increasingly clear that human tumors are incredibly complex, and identifying molecular drivers of carcinogenesis among the larger number of background events has proven difficult. Comparative analysis of the information gained from human tumor profiling with data from animal models provides an improved ability to distinguish driver events contributing to human disease.

The Sleeping Beauty (SB) transposon mutagenesis system has proven useful for identifying drivers of tumorigenesis in a wide variety of tissue types [4]. We have previously used SB mutagenesis to generate mice that developed HCC [5]. Subsequent genetic analysis of SB-induced liver tumors identified the *Dlk1-Dio3* imprinted domain as a common target of transposon-induced mutations. This highly complex domain contains genes encoding protein-coding transcripts, long non-coding RNAs (lncRNAs), microRNAs (miRNAs), and small nucleolar RNAs (snoRNAs). Expression of domain members is regulated in an allele-specific manner and depends on epigenetic modifications established in the germline [6]. Regulation of this expression pattern is maintained, at least in part, by multiple differentially methylated regions (DMRs) throughout the domain that are methylated on the paternally inherited allele. Maintenance of imprinting is critical for normal function, as evidenced by the fact that uniparental disomy (UPD) for either parental allele leads to severe and widespread developmental defects in both mouse models [7] and human patients [8].

A link between the *Dlk1-Dio3* domain and HCC has previously been identified. Interestingly, it has been reported that adeno-associated viral (AAV) vector integration within the same region of the domain as the SB transposon integrations in our model is associated with HCC development in mice [9], [10]. AAV integrations were found to alter expression of several domain members, preventing elucidation of a clear molecular mechanism of tumorigenesis. Other studies have also identified correlation between disrupted expression from the *Dlk1-Dio3* domain and HCC [11]–[15], often with several domain members showing aberrant expression. The majority of these studies are correlative in nature, and no attempt is made to validate tumorigenic function of domain members through direct modulation of gene expression.

Here we describe a series of experiments that initially utilized deep-sequencing analyses to obtain detailed gene expression profiles of the SB-induced HCCs. This approach revealed that transposon integration within the *Dlk1-Dio3* domain has variable effects on expression of several elements throughout the imprinted domain, but uniformly drives dramatic overexpression of *Retrotransposon-like 1* (*Rtl1*). Validation experiments demonstrate that hepatic overexpression of *Rtl1* promotes tumorigenesis *in vivo*. Additionally, we find that *RTL1* is aberrantly expressed in ∼30% of human HCC samples, suggesting that it may be a relevant therapeutic target.

*Rtl1* is a poorly characterized gene that encodes a predicted transmembrane protein with aspartic protease activity. Interestingly, this locus is derived from domestication of a sushi-ichi-related retrotransposon [16] and is unique to placental mammals [17]. This study identifies *Rtl1* as a novel oncogene involved in hepatocarcinogenesis and suggests that its expression may be used as a prognostic indicator and/or targeted therapeutically to improve outcome for patients with HCC. It also represents one of the first direct *in vivo* demonstrations of a role for a co-opted genetic element in driving carcinogenesis.

## Results/Discussion

### Determining the effect of transposon integration on *Dlk1-Dio3* domain members

**Figure 1:**
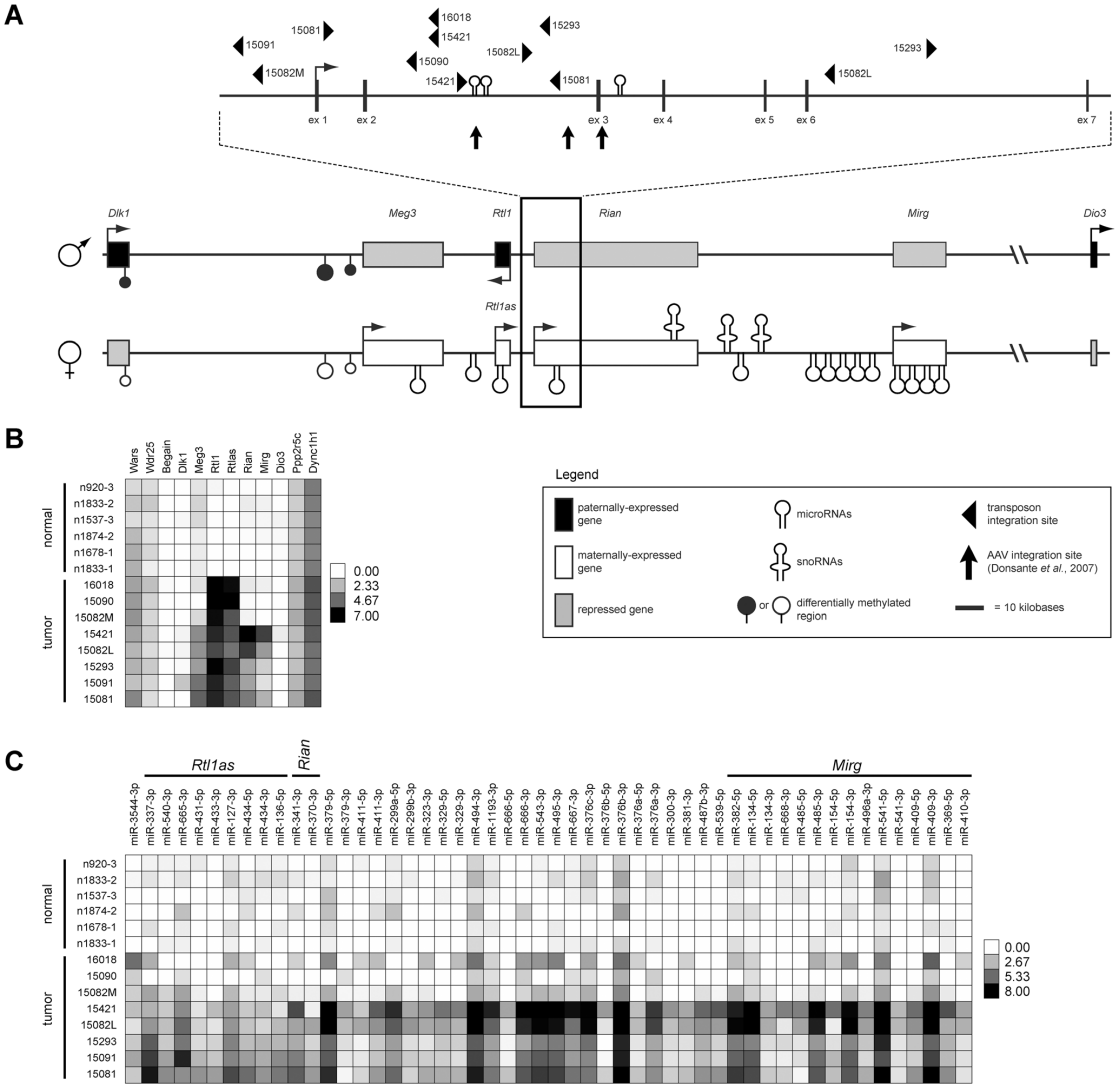
*Dlk1-Dio3* domain transposon integration sites in SB–induced HCC and effects on domain expression. (A) The *Dlk1-Dio3* imprinted domain spans ∼800 kilobases at the distal end of mouse chromosome 12 (human chr14q32). Three protein-coding genes are expressed from the paternal allele (*Dlk1*, *Rtl1*, and *Dio3*). The maternal allele encodes four lncRNAs (*Meg3*, *Rtl1as*, *Rian*, and *Mirg*), as well as several miRNAs and snoRNAs. SB transposon and AAV integration sites found to be associated with HCC development in mice are depicted. (B) Intensity plot showing normalized expression levels of long transcripts within and surrounding the *Dlk1-Dio3* domain in SB-induced HCCs and normal livers. (C) Intensity plot showing normalized expression of *Dlk1-Dio3* domain miRNAs. miRNAs contained within lncRNAs are indicated. miRNAs with no detected expression across all samples were omitted.

**Figure 2:**
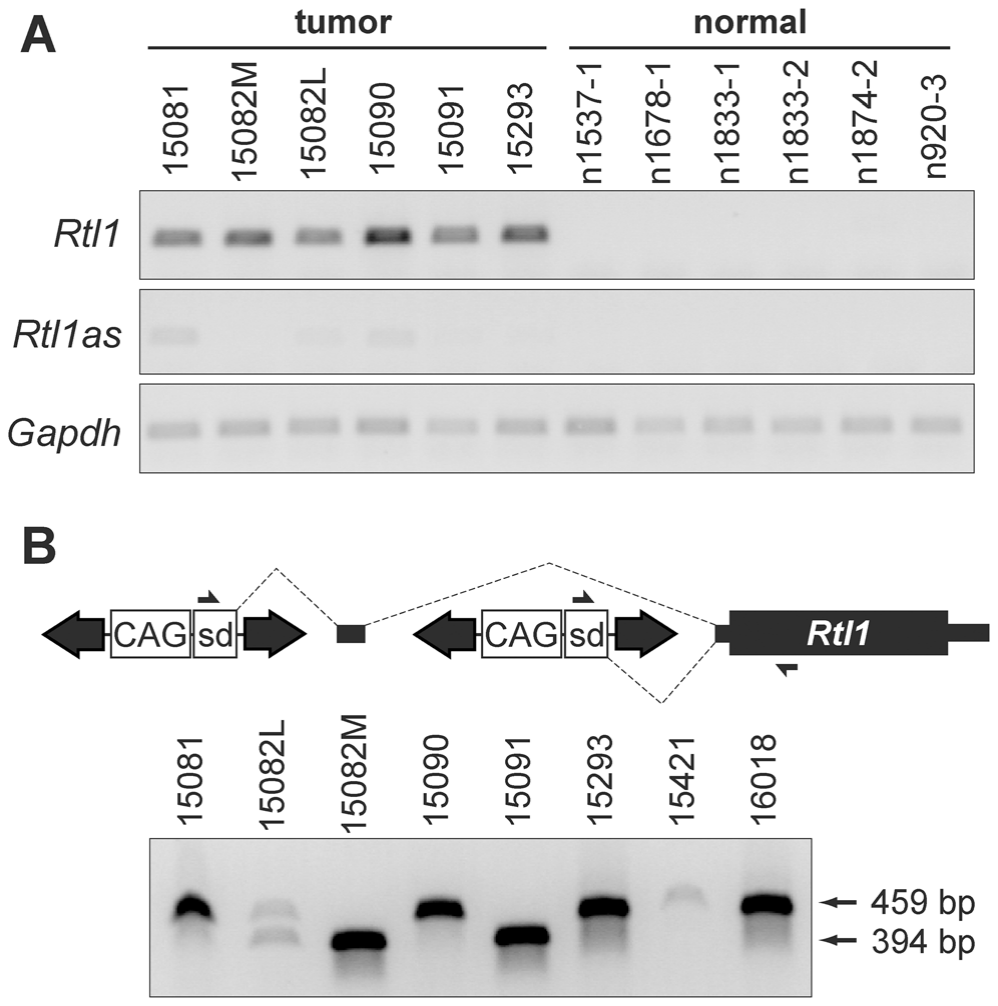
Integrated transposons drive overexpression of *Rtl1*. (A) Strand-specific RT-PCR detected activation of *Rtl1* expression in SB-induced HCCs, with minimal activation of *Rtl1as* observed. No expression of either transcript was detected in normal liver. (B) Transposons integrated upstream of *Rtl1* drive its expression by generating fusion transcripts. Transcription initiated from the CAG promoter within the transposon splices into *Rtl1*, either directly or via inclusion of an upstream cryptic exon. PCR to detect fusion transcripts was performed on cDNA from SB-induced HCCs using the indicated primers. Transposon-driven expression of *Rtl1* was detected for all of the tumor samples harboring *Dlk1-Dio3* domain integrations. sd, splice donor.

We previously reported the identification of a 33 kilobase region of the imprinted *Dlk1-Dio3* domain as a common target of transposon insertion in an SB-induced model of HCC [5] (Figure 1A). Given the domain's complexity and previous studies demonstrating altered expression of multiple domain members in response to insertion of exogenous DNA [9], [10], [18], we used both transcriptome and miRNA sequencing approaches to obtain expression profiles of eight SB-induced HCCs with *Dlk1-Dio3* integrations and six normal livers for comparison (Figure 1B–1C, Figures S1 and S2, Tables S1 and S2). Expression of *Dlk1-Dio3* domain miRNAs was low to undetectable in normal liver. Similar results were detected for three of eight tumors, while the remaining five tumors displayed activated expression of several imprinted miRNAs. Thus, transposon insertion in the *Dlk1-Dio3* domain does not consistently alter miRNA expression. Interestingly, tumor samples with elevated expression of imprinted miRNAs also showed enhanced expression of *Meg3* and *Rian*, suggesting a possible transposon-mediated loss of imprinting effect. Dramatic activation of expression from the locus encoding *Rtl1* and *Rtl1 antisense* (*Rtl1as*) was observed in all eight SB-induced HCCs, while no significant expression was detected in normal liver. Notably, elevated expression from this locus is the only event that was consistently observed in all SB-induced HCCs with *Dlk1-Dio3* integrations (Figure 1B–1C). Because transcription can occur on either strand at this locus [19], strand-specific RT-PCR was performed to determine whether the observed increase resulted from expression of *Rtl1*, *Rtl1as*, or a combination of both transcripts. As shown in Figure 2A, reads from the locus encoding *Rtl1* and *Rtl1as* detected in HCCs were derived primarily from transcription of the protein-coding sense strand (*i.e. Rtl1*). The lack of detectable *Rtl1* in normal liver suggests that transposon integration results in activation of a normally transcriptionally silent allele.

### Integrated transposons directly drive *Rtl1* expression

As we previously reported, SB transposon integration sites in HCC samples clustered near the 5′ end of *Rian* within the *Dlk1-Dio3* domain [5]. Our initial characterization of transposon integrations was performed using ligation-mediated (LM)-PCR followed by pyrosequencing. It has been shown that this approach yields suboptimal sequencing depth for confident identification of clonal insertion sites [20]. To ensure adequate sequence coverage, the SB-induced HCCs were re-sequenced for the current study using the Illumina platform. Surprisingly, while integrations near the 5′ end of *Rian* were still found to be the most common event, a transposon orientation bias was revealed that had not previously been evident. For many of the tumors, multiple transposon integrations were identified in this region, and for each of the tumors at least one of these integrations was in the same orientation as *Rtl1* (Figure 1A).

To validate the significance of transposon integrations upstream of *Rtl1* in SB-induced HCCs, insertion sites from a larger set of tumors, as well as some normal livers (Rogers et al., in press), were sequenced using the Illumina platform. A quantitative analysis of all transposon integrations in the *Dlk1-Dio3* domain for these samples is provided in Figure S3. Consistent with recent studies demonstrating minimal insertion bias for SB transposon integration [21], [22], background insertion sites identified in normal liver and subclonal insertions in HCC samples did not show any evidence for preferential integration within the *Dlk1-Dio3* domain. In contrast, clonal sites identified in tumors were highly enriched upstream of *Rtl1*, suggesting positive selection for insertions in this region during the process of tumorigenesis. This analysis further confirmed that transposon integrations in the same transcriptional orientation as *Rtl1* are preferentially detected specifically in HCCs. Based on these results, we hypothesized that the high levels of *Rtl1* observed in tumors were driven directly by transposons integrated upstream. Amplification of transposon/*Rtl1* fusion products from cDNA confirmed transposon-driven *Rtl1* overexpression for each of the tumors harboring integrations in this region (Figure 2B). Two different sizes of fusion products were detected, representing direct splicing of the T2/Onc3 transposon into *Rtl1* (smaller product) or inclusion of a cryptic upstream exon (larger product). Importantly, both fusion products encode the full *Rtl1* open reading frame and are thus predicted to drive overexpression of functional Rtl1 protein.

Two additional Sleeping Beauty screens have been reported in which liver tumors were generated and characterized [23], [24]. Neither of these studies identified the *Dlk1-Dio3* domain as a common site of integration. Both screens utilized T2/Onc mice as the source of mutagenic transposons. This transposon is similar in structure to that of the T2/Onc3 strain used in our study, but a distinct promoter is included within the transposon. T2/Onc transposons contain the murine stem cell virus (MSCV) 5′ long-terminal repeat (LTR) promoter, while T2/Onc3 transposons contain the cytomegalovirus (CMV) enhancer/chicken β-actin (CAG) promoter. Differences in promoter activities likely affect the profile of mutations that are selected for in tumors resulting from SB mutagenesis. We suspect that the MSCV promoter may be too weak to overcome the influence of imprinting within the *Dlk1-Dio3* domain to drive sufficient hepatic *Rtl1* expression to provide cells with a selective advantage and promote tumorigenesis. The CAG promoter, which has a much higher activity in epithelial cells like hepatocytes, may be better able to drive *Rtl1* overexpression when integrated upstream, resulting in frequent selection of cells with such mutations in tumors. Consistent with this idea, insertional mutations upstream of *Rtl1* have been linked to liver tumor development in two independent studies that utilized viral vectors containing promoters with high activity in hepatocytes [9], [15].

### Rtl1 expression in cultured hepatocytes promotes growth in ECM

Our RNA profiling analyses and fusion transcript detection led us to conclude that the primary tumor-driving event under positive selection in SB-induced HCCs is activation of *Rtl1*. While we cannot exclude the possibility that other domain members play a role independently and/or cooperatively with *Rtl1*, in our model it seems to be the dominant driver of hepatocarcinogenesis. It should be noted that other models of HCC have been described in which altered expression of maternal *Dlk1-Dio3* domain members is observed in the absence of *Rtl1* activation [25], suggesting that distinct roles may exist for both paternal and maternal components of the domain in different subtypes of HCC.

**Figure 3:**
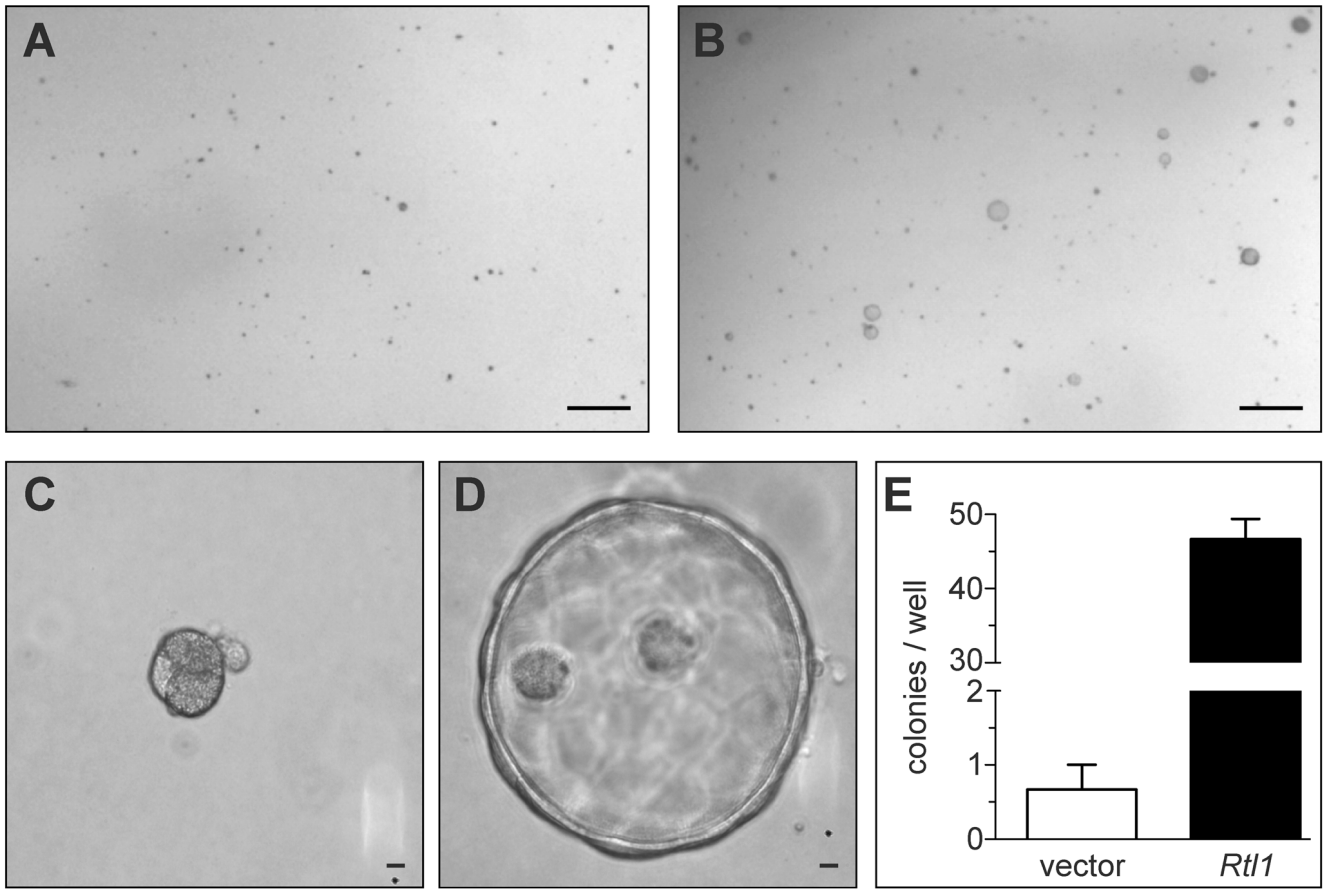
Rtl1 promotes growth of cultured hepatocytes in extracellular matrix. Two weeks after plating cultured hepatocytes in a matrix of Matrigel, cells transfected with an empty vector construct (A) failed to grow significantly. Cells transfected with an *Rtl1* expression construct (B) grew to form several large colonies. (C–D) Rtl1 promotes growth of large cyst-like structures. Increased magnification reveals that cells lacking Rtl1 (C) form small, dense colonies, while those expressing Rtl1 (D) form large cyst-like colonies composed of several cells. (E) Quantification of colonies per well formed by each cell line in a 24-well plate. The results depicted are based on three experimental replicates per condition and are representative of experiments conducted on three separate days. Scale bars = 0.5 cm (A–B) and 100 µm (C–D).

To study the effects of *Rtl1* overexpression on hepatocyte growth and morphology *in vitro*, we stably overexpressed it in the murine hepatocyte cell line TIB-73. Importantly, this cell line is non-tumorigenic and lacks endogenous expression of *Rtl1*. Based on the predicted protein structure of Rtl1, which contains an extracellular protease domain, we hypothesized that its effects may be mediated via cleavage of a substrate within the extracellular matrix (ECM). To test this hypothesis, TIB-73 cells expressing either *Rtl1* or an empty vector were embedded in a matrix of Matrigel, plated in 24-well plates, and cultured in serum-free medium. Two weeks after plating, cells expressing *Rtl1* had grown to form dozens of cyst-like colonies composed of several cells (Figure 3B, 3D). In contrast, cells lacking *Rtl1* expression formed less than one colony per well on average, and colonies that did form were much denser and smaller (Figure 3A, 3C). These results demonstrate that *Rtl1* expression promotes growth of hepatocytes in the presence of ECM in the context of physiologically relevant levels of growth factors, and they are consistent with our hypothesis that Rtl1 acts by cleaving an ECM component. ECM is an important aspect of the tumor microenvironment, particularly in the liver. The process of liver fibrosis, which involves ECM remodeling and expansion, is strongly linked to HCC, with nearly 90% of cases developing in this context [26]. One mechanism by which fibrosis may contribute to the development of HCC is through sequestration of growth factors in the newly remodeled ECM [27]. According to this model, subsequent release of growth factors through protease-mediated cleavage of ECM components promotes proliferation of adjacent hepatocytes. Our results suggest that Rtl1 may contribute to hepatocarcinogenesis via this mechanism.

### *In vivo* hepatic Rtl1 expression drives tumorigenesis

**Figure 4:**
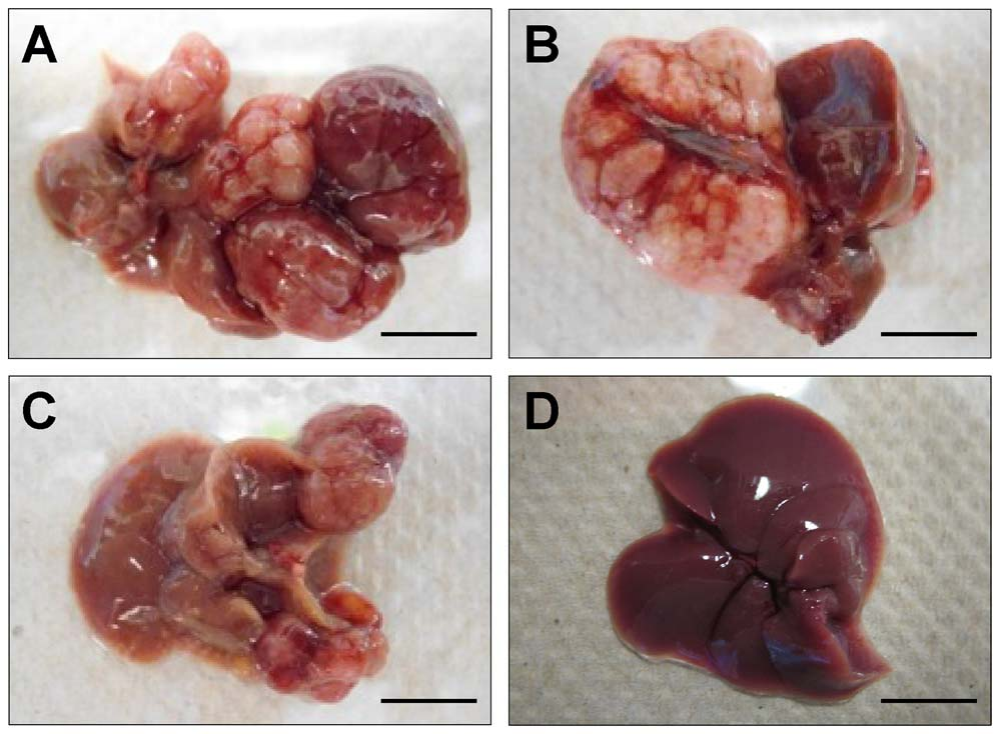
*In vivo* hepatic overexpression of *Rtl1* promotes tumorigenesis. (A–C) Macroscopic images of tumor-containing whole livers from mice injected with *Rtl1* overexpression constructs via hydrodynamic tail vein injection. Mice were euthanized and livers collected nine months post-injection. (D) A normal liver from a hydrodynamically injected mouse is shown for comparison. Scale bars = 1 cm.

We next sought to determine if *Rtl1* overexpression is sufficient to promote hepatocarcinogenesis *in vivo*. Mice with stable hepatic expression of *Rtl1* were generated by hydrodynamic tail vein injection of transposon-based expression constructs [28] into *Fah*-deficient male mice expressing SB transposase [24]. Selective repopulation of the liver was achieved through inclusion of a separate *Fah* expression vector that allowed stably transfected cells to survive withdrawal of NTBC [29], an event that triggers the death of *Fah*-null hepatocytes. Mice were euthanized nine months post-injection to assess liver tumorigenesis. Of fourteen mice injected with *Rtl1* overexpression constructs, twelve (86%) developed liver tumors, with an average of 2.9 tumors per mouse (Table 1, Figure 4). In another experimental condition, a third construct encoding a short hairpin directed against *Trp53* was additionally included. Loss of p53 function is one of the most commonly observed molecular abnormalities in human HCC, occurring in ∼30% of cases and making this a relevant context in which to validate putative oncogenes. Of twelve mice injected with all three transposon constructs, ten (83%) developed liver tumors, with an average of 4.3 tumors per mouse. Six of the mice from this cohort were sacrificed at time points earlier than nine months. When considering only those mice that were aged for nine months to allow direct comparison between the two experimental groups, five of six (83%) mice with p53 knockdown in addition to *Rtl1* overexpression developed liver tumors, with an average of 6.7 tumors per mouse. This is significantly higher (p = 0.027) than the number of tumors per mouse developed with *Rtl1* overexpression alone. Knockdown of p53 in tumors was assessed by western blot (Figure S4A). Although efficiency was somewhat variable, the majority of tumors showed significant knockdown.

**Table 1:** Liver tumors developed in hydrodynamically injected mice.

Mouse ID	Experimentalgroup	Days post-injection	Liver nodules
M1381	Rtl1 only	240	4
M1382	Rtl1 only	240	1
M1383	Rtl1 only	240	0
M1384	Rtl1 only	240	6
M1375	Rtl1 only	251	1
M1376	Rtl1 only	251	3
M1482	Rtl1 only	251	5
M1483	Rtl1 only	251	1
M1484	Rtl1 only	251	4
M1485	Rtl1 only	251	3
M1491	Rtl1 only	251	5
M1492	Rtl1 only	251	4
M1501	Rtl1 only	251	0
M1502	Rtl1 only	251	3
		**Avg**	**2.86**
M1371	Rtl1+shp53	120	0
M1372	Rtl1+shp53	120	2
M1373	Rtl1+shp53	120	2
M1374	Rtl1+shp53	120	1
M1441	Rtl1+shp53	184	2
M1442	Rtl1+shp53	184	5
		**Avg**	**2.00**
M1391	Rtl1+shp53	251	5
M1392	Rtl1+shp53	251	0
M1394	Rtl1+shp53	251	6
M1451	Rtl1+shp53	254	5
M1454	Rtl1+shp53	254	16
M1455	Rtl1+shp53	254	8
		**Avg**	**6.67**

It has been shown that following liver repopulation, the *Fah* mouse model is predisposed to tumor formation in the absence of any additional transgene [30], [31]. The tumors that develop in this context uniformly lack expression of *Fah*. We assessed expression of both *Rtl1* and *Fah* by RT-PCR in fourteen tumors developed following hydrodynamic injection (Figure S4B). Of these fourteen tumors, eleven were found to express both genes. This result suggests that while a small subset of our tumors are likely background events developed independently of *Rtl1* expression due to the model's predisposition, the majority of tumors were induced directly by overexpression of *Rtl1*. Further evidence for the tumorigenic activity of *Rtl1 in vivo* comes from a recently published study showing that liver tumors develop in mice following hepatic lentiviral delivery [15].

### *RTL1* activation in human HCC

**Figure 5:**
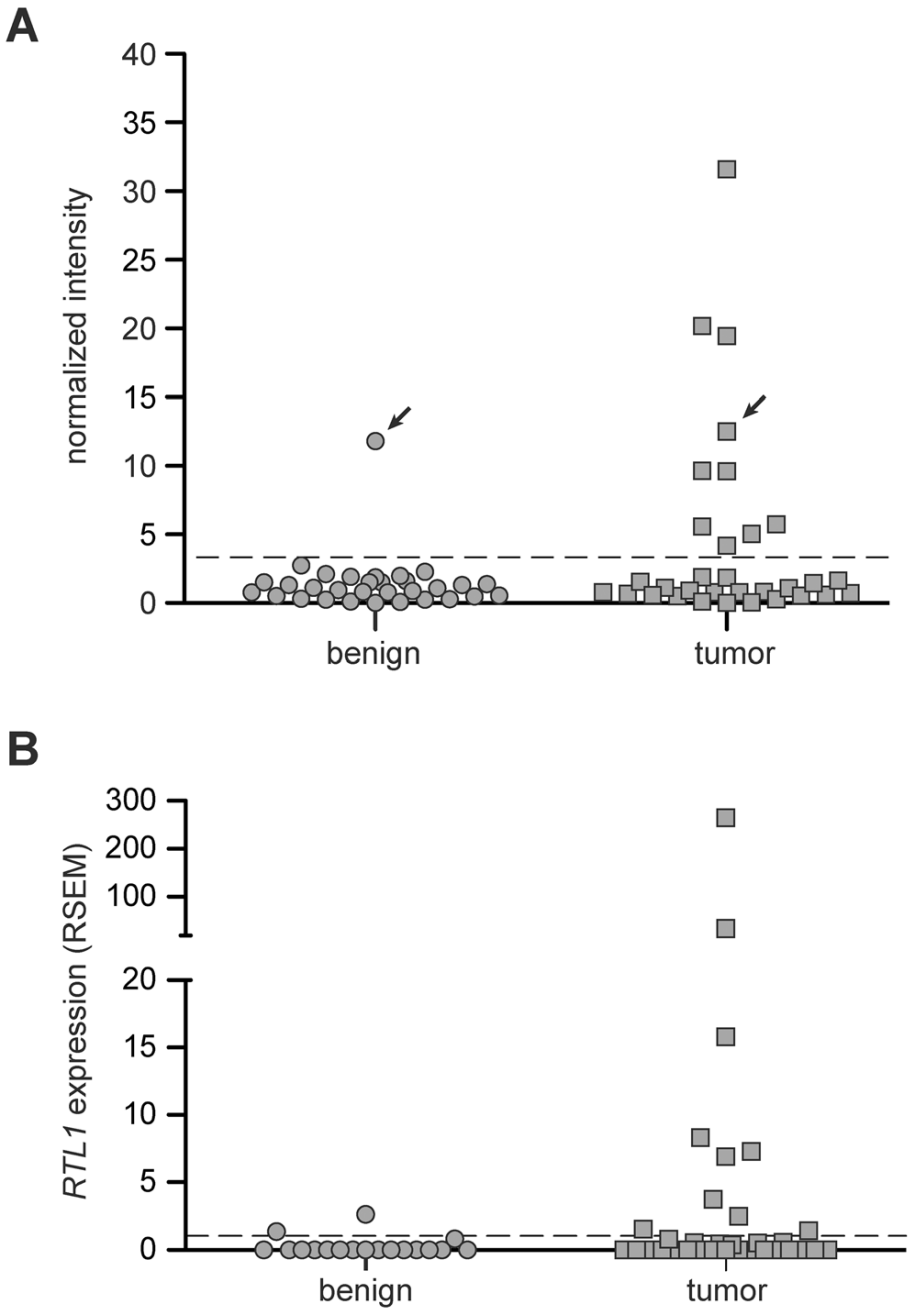
Expression of *RTL1* in human HCC. (A) *RTL1* expression in human HCC and matched benign liver samples was analyzed by RT-PCR. Plotted values represent normalized band intensities from imaged gels. The threshold above which a sample was scored as positive for significant *RTL1* expression (dashed line) was set at three standard deviations above the average intensity value in benign samples lacking detectable expression. For the one patient with significant *RTL1* expression detected in benign tissue, the matched HCC sample also displayed expression (indicated with arrows). (B) Plot of *RTL1* expression in human HCC and normal liver samples based on RNASeq data available through TCGA. The threshold above which a sample was scored as positive for significant *RTL1* expression (dashed line) was set at one standard deviation above the average expression level in tumor-free liver. RSEM, RNASeq by Expectation Maximization.

In order to determine the prevalence of *RTL1* activation in human disease, RT-PCR was performed on a collection of thirty-three human HCC RNA samples, along with matched benign adjacent liver tissue (Figure 5A, Figure S5). A lack of significant expression was observed for all but one of the benign liver samples. In contrast, significant activation of *RTL1* was detected in 30% (10/33) of analyzed tumors. To assess *RTL1* expression in another set of human HCCs, we utilized RNASeq data available through The Cancer Genome Atlas (TCGA) consortium. Consistent with our initial analysis, *RTL1* expression was found to be significantly activated in 30% (10/33) of analyzed tumors (Figure 5B). Low-level expression was detected in two of the adjacent benign tissue samples for which sequence data was available. It should be noted that four of the tumor samples included in the TCGA dataset overlap with the initial set of 33 samples analyzed by RT-PCR. No expression of *RTL1* was detected in these four samples by either analysis. A notable gender disparity is observed in human HCC, wherein men are around three times more likely to develop the disease than women [1]. We analyzed our human expression data to determine if *RTL1* overexpression was associated with tumors from one gender or the other, but failed to detect evidence of any bias. Based on the combined set of human samples that we analyzed, *RTL1* was found to be overexpressed in samples from 12/38 males (32%) and 8/24 females (33%).

Unfortunately, there is very little existing data on the expression of *RTL1* in disease states, including cancer. Most expression analyses utilize commercially available microarray platforms, the vast majority of which lack probes for *RTL1*. While multiple studies have identified correlative links between disrupted expression of other *Dlk1-Dio3* domain members and HCC [9]–[14], expression of *RTL1* has not typically been assessed. This may be due in part to the fact that *RTL1* is a single exon gene, preventing straightforward design of primers that specifically amplify from cDNA and not genomic DNA. Notably, we have utilized a method for *RTL1* expression analysis that adds a unique sequence tag during reverse-transcription [32], thus allowing specific amplification from cDNA and eliminating background amplification from genomic DNA.

In the setting of spontaneous hepatocarcinogenesis in humans, *RTL1* activation may occur as a result of loss of imprinting (LOI) within the *Dlk1-Dio3* domain. Epigenetic abnormalities are known to play a large role in driving tumor development and progression, in part through induction of LOI [33]. A direct causal role for LOI in cancer was demonstrated by Holm *et al.*, who showed that chimeric mice created using embryonic stem cells lacking imprinting-specific DNA methylation develop multiple tumor types with nearly complete penetrance [34]. The most common tumor type observed was HCC, suggesting that LOI in the liver confers a strong predisposition to cancer. While expression from the *Dlk1-Dio3* domain was not examined in the study, the results we present here suggest that hepatic activation of *Rtl1* may be a driving factor in the HCCs that were developed. Interestingly, Wang *et al.* reported loss of methylation within the *Rtl1* locus in mouse HCCs resulting from AAV integration [10], although effects on *Rtl1* expression were not determined. To assess whether or not *Rtl1* overexpression is associated specifically with altered expression of other imprinted genes in our SB-induced HCCs, analysis of variance (ANOVA) was conducted on the whole transcriptome to identify genes with differential expression between *Rtl1*-overexpressing tumors and normal liver. Following Bonferroni correction, 3 of 125 imprinted genes and 474 of 20,707 non-imprinted genes were identified as having significantly different expression between the two sample sets. By Fisher's exact test, these proportions are not significantly different (p = 0.760). This analysis shows that activation of *Rtl1* does not correlate specifically with altered expression of other imprinted genes in our tumors.

### *Rtl1*-expressing mouse HCCs resemble human S1 subclass

**Figure 6:**
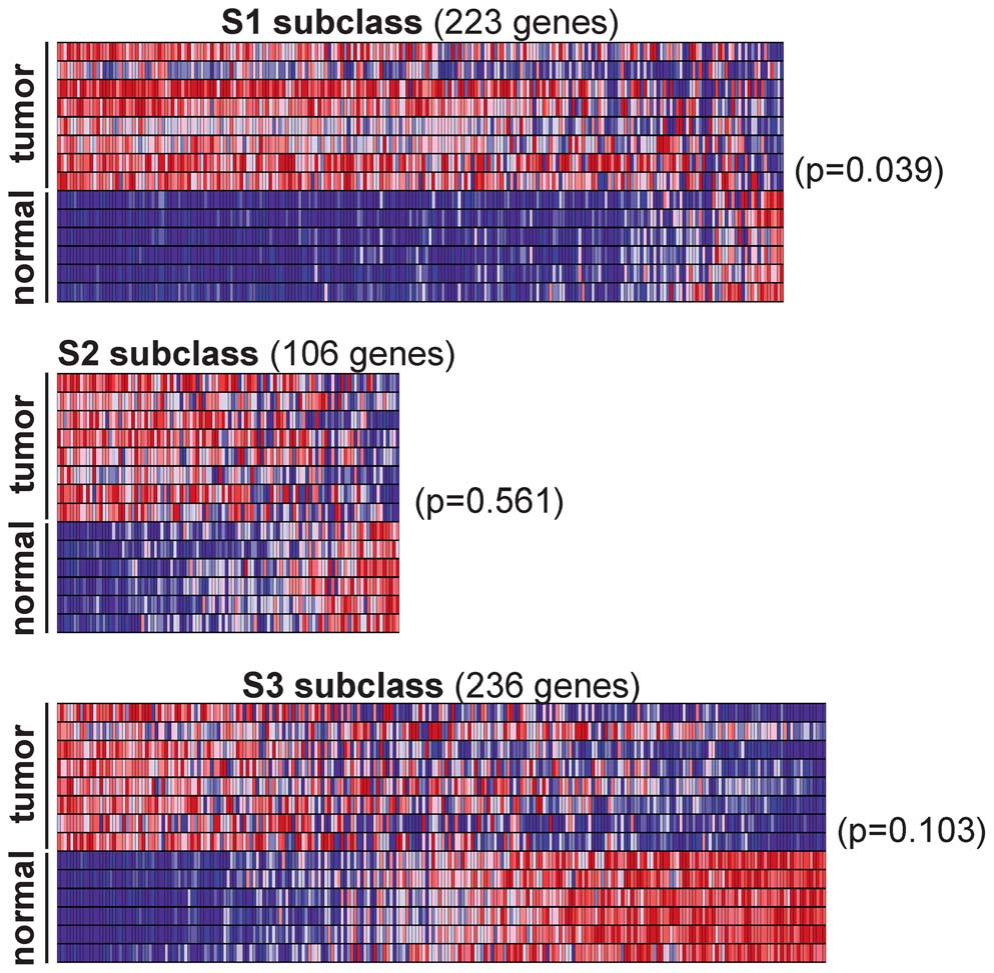
*Rtl1*-expressing mouse HCCs resemble human S1 subclass. Expression levels for the gene sets defining human HCC subclasses S1, S2, and S3 were analyzed in SB-induced HCCs and normal livers. Gene Set Enrichment Analysis (GSEA) was conducted for each subclass independently to assess the significance of differential expression between tumor and normal samples. Heat maps generated by GSEA are shown. This analysis revealed a significant (p = 0.039) overexpression of the genes defining human subclass S1 in SB-induced HCCs, as compared to normal liver.

Next we sought to determine if *Rtl1*-induced HCCs in mice resemble a specific subtype of human HCC. An integrative meta-analysis of human HCC gene expression profiles has identified three major expression subtypes called S1, S2, and S3 [35]. Transcriptome sequencing data from the mouse HCCs overexpressing *Rtl1* was used to determine the extent to which these SB-induced tumors resemble human HCC. Expression levels of genes defining the S1, S2, and S3 subclasses of human HCC were assessed for each of the SB-induced tumors and normal liver samples. Unsupervised clustering of samples based on expression of constituent genes was performed individually for each subclass. The results show that the SB-induced tumors resemble human HCCs within the S1 subclass (Figure 6). This was further supported by Gene Set Enrichment Analysis (GSEA) [36], [37] that showed a statistically significant association (p = 0.039) between *Rtl1*-induced HCCs and the S1 expression class. Immunohistochemistry was performed to validate protein expression of two S1 subclass genes in SB-induced HCC (Figure S6). This subclass of human HCC is associated with poor to moderate cellular differentiation, activation of the WNT signaling pathway, and early tumor recurrence.

### Potential of *RTL1* as a therapeutic target and/or biomarker

*Rtl1* is a poorly characterized gene that encodes a predicted transmembrane protein with aspartic protease activity. Knockout studies in mice have demonstrated a role in the placental feto-maternal interface [38], but functional studies in other tissues are lacking. Experiments to determine the necessity of Rtl1's protease domain for its ability to promote tumorigenesis and to identify targets of its activity will help to clarify the oncogenic mechanism. If required, RTL1's protease activity represents a promising target for therapeutic intervention in HCC patients. Pepstatin is a naturally occurring bacterial peptide that demonstrates broad potential to inhibit aspartic proteases [39]. Additionally, more specific inhibitors have successfully been developed that target the activity of other aspartic proteases, including renin [40] and HIV-1 protease [41]. It is also possible that *RTL1* expression could be a useful biomarker for HCC. Based on the human samples that we analyzed, its expression appears to be highly tumor-specific. Although low-level expression was detected in three non-tumor liver samples, all of the benign samples came from HCC patients and are therefore unlikely to be representative of truly normal liver.

### Conclusion

In this study we identify *Rtl1*, a co-opted imprinted gene, as a novel driver of hepatocarcinogenesis. Mutations resulting in its overexpression were highly selected for in liver tumors developed using a forward genetic screen. While several correlative results linking the *Dlk1-Dio3* domain to HCC development have been reported, our study provides direct evidence that modulation of a domain member *in vitro* and *in vivo* promotes a tumorigenic phenotype. We show here that overexpression of *Rtl1* in cultured hepatocytes results in an increased growth ability in extracellular matrix. We also show that overexpression via hydrodynamic gene delivery results in highly penetrant liver tumor formation in mice. Additionally, a subset of human HCCs displays overexpression of *RTL1*, suggesting it may be a relevant therapeutic target for patients.

## Materials and Methods

### Mice

SB-induced mouse HCCs used in this study were generated as previously described [5]. All tumors used in this study came from male mice and were collected using procedures approved and monitored by the Institutional Animal Care and Use Committees at the National Cancer Institute-Frederick and the University of Minnesota.

### Human tissue samples

Paired tumor and benign liver tissues were obtained from 33 patients undergoing resections for HCC at Mayo Clinic between 1987 and 2003, snap-frozen in liquid nitrogen, and stored at −80°C. The Mayo Clinic Institutional Review Board approved the study.

### RNA sequencing and data analysis

#### Transcriptome sequencing

Total RNA was collected from SB-induced HCC and normal liver samples using the miRNeasy kit (Qiagen). Library preparation and sequencing were performed using Illumina's mRNA-Seq workflow. For data normalization, the raw number of reads for each transcript was converted to reads per kilobase per million mapped reads (RPKM) [42]. This was followed by log_2_ transformation of the RPKM value +1. Unsupervised clustering was performed on samples based on normalized expression of genes with variation in Euclidean distance among samples of at least 2.5 standard deviations using Cluster 3 software [43]. Heat maps were generated using Java TreeView software [44].

#### miRNA sequencing

Total RNA was collected from SB-induced HCC and normal liver samples using the miRNeasy kit (Qiagen). The flashPAGE Fractionator system (Life Technologies) was used to isolate RNAs shorter than 40 nt. Library preparation and sequencing were performed using the SOLiD small RNA expression workflow (Life Technologies). For data normalization, the raw number of reads for each miRNA was converted to reads per 100,000 mapped reads. This was followed by log_2_ transformation of the normalized value +1. Unsupervised clustering was performed on samples based on normalized expression of genes with variation in Euclidean distance among samples of at least 1.5 standard deviations using Cluster 3 software [43]. Heat maps were generated using Java TreeView software [44].

### RT–PCR

#### Strand-specific RT–PCR to detect expression of Rtl1 and Rtl1as

One nanogram total RNA was used as template for cDNA synthesis with AMV reverse transcriptase (New England Biolabs). The cDNA synthesis reaction was primed with oligonucleotides complementary to *Gapdh* (Gapdh_R: 5′ – TGTAGGCCATGAGGTCCACCAC – 3′) and either *Rtl1* (Rtl1_R: 5′ – GGAGCCACTTCATGCCTAAGACGA – 3′) or *Rtl1as* (Rtl1as_R: 5′ – GTGGAGAACTTCGCTGTCATCGC – 3′). PCR was performed with primers to detect transcripts for *Gapdh* (Gapdh_F: 5′ – TTGTCTCCTGCGACTTCAA – 3′ and Gapdh_R (amplicon 150 bp)), *Rtl1* (Rtl1_F: 5′ – TACTGCTCTTGGTGAGAGTGGACCC – 3′ and Rtl1_R (amplicon 297 bp)), or *Rtl1as* (Rtl1as_F: 5′ – TCTCCACTCGAGGGTACTCCACCT – 3′ and Rtl1as_R (amplicon 298 bp)).

#### Transposon/Rtl1 fusion transcript detection

One microgram total RNA was used as template for oligodT-primed cDNA synthesis with Superscript III reverse transcriptase (Life Technologies). Control reactions lacking the RT enzyme were also performed. PCR was performed with a forward primer within the transposon splice donor (SD_F: 5′ – AAGCTTGCTACTAGCACCAGAACGCC – 3′) and reverse primer within *Rtl1* (Rtl1_R2: 5′ – TTCCTGGGCTGGGCCACTATC – 3′) (amplicon 394 bp or 459 bp, depending on splicing pattern).

#### Detection of RTL1 in human HCCs

Five hundred nanograms total RNA was used as template for cDNA synthesis with AMV reverse transcriptase (New England Biolabs). The cDNA synthesis reaction was primed with oligonucleotides complementary to *TBP* (TBP_R: 5′ – GCCATAAGGCATCATTGGAC – 3′) and *RTL1* (RTL1_RT_tag: 5′ – GTAATACGACTCACTATAGGGCCTCGATAGGGGAGATGTTGC – 3′). The RTL1_RT_tag primer adds a unique 22 base sequence (underlined) to the 5′ end of the newly synthesized cDNA. Control reactions lacking the RT enzyme were also performed. PCR was performed with primers to detect transcripts for *TBP* (TBP_F: 5′ – GCTGAGAAGAGTGTGCTGGA – 3′ and TBP_R (amplicon 204 bp)) and *RTL1* (RTL1_F: 5′ – TTCTACTGGGGAGTCGAGGA – 3′ and RT_tag_R: 5′ – GTAATACGACTCACTATAGGGC – 3′ (amplicon 238 bp)). RT_tag_R binds to the unique sequence tag added during cDNA synthesis, minimizing the potential for amplification from contaminating genomic DNA. Formamide was included in the PCR mix at a final concentration of 3% or 5% for amplification of *TBP* or *RTL1*, respectively. Gel images were processed using VisionWorks®LS image analysis software (UVP) to obtain intensity values for each lane. For each sample, values were normalized by subtracting the - RT lane from the +RT lane, then dividing each corrected value by the average corrected intensity value in benign samples lacking detectable expression.

#### Detection of *Rtl1* and *Fah* in tumors induced by hydrodynamic injection

One microgram total RNA was used as template for oligodT-primed cDNA synthesis with Superscript III reverse transcriptase (Life Technologies). Control reactions lacking the RT enzyme were also performed. PCR was performed with primers to detect *Rtl1* (Rtl1_F2: 5′ – GTGGAGAACTTCGCTGTCATCGC – 3′ and Rtl1_R2: 5′ – TCTCCACTCGAGGGTACTCCACCT – 3′ (amplicon 298 bp)), *Fah* (Fah_F: 5′ – CTTCTGCGACAATGCACCT – 3′ and Fah_R: 5′ – ACCACAATGGAGGAAGCTCG – 3′ (amplicon 172 bp)), or *Tbp* (Tbp_F: 5′ – CTATCACTCCTGCCACACCA – 3′ and Tbp_R: 5′ – CAGTTGTCCGTGGCTCTCTT – 3′ (amplicon 189 bp)).

### Illumina sequencing of transposon insertions

DNA from SB-induced tumors was prepared for sequencing of transposon integration sites as previously described [20].

### Matrigel growth assay

Stable cell lines were generated by delivery of piggyBac transposon constructs encoding either *Rtl1* or an empty vector into TIB-73 (ATCC: BNL CL.2) cultured mouse hepatocytes. 24-well plates were coated with a thin layer of Matrigel basement membrane mix (BD Biosciences) and allowed to set up for 30 minutes at 37°C. For each stable cell line, cells were trypsinized and washed with PBS before resuspension of 5,000 cells in additional Matrigel. The resuspended cells were plated on top of the thin layer of basement membrane mix and allowed to set up, followed by addition of serum-free, low-glucose DMEM (Life Technologies). Images were taken two weeks after plating.

### Hydrodynamic gene delivery

Hydrodynamic tail vein injection into *Fah*-deficient male mice expressing SB*11* transposase was performed as previously described [24]. A plasmid expressing *Rtl1* from the human *PGK* promoter and flanked by SB transposon inverted repeat/direct repeats (IR/DRs) was generated by amplifying the open reading frame of *Rtl1* from C57Bl/6J mouse genomic DNA and subcloning it into pT2/PGK-pA. This plasmid was co-injected with PT2/PGK-FAHIL, a plasmid containing an SB IR/DR-flanked expression cassette for *Fah* and firefly luciferase. Some mice were additionally injected with pT2/shp53, a plasmid containing an SB IR/DR-flanked expression cassette for a short-hairpin RNA directed against *Trp53* [29], [45].

### Western blotting

Total protein was collected from liver tumor samples by homogenization in RIPA lysis buffer. Samples were boiled for five minutes in a reducing buffer and SDS-PAGE was performed. Proteins were transferred to nitrocellulose membranes for blotting. Primary antibodies used were anti-p53 (Cell Signaling Technology #2524), anti-GFP (Clontech #632380), and anti-β-tubulin (Sigma-Aldrich #T4026).

### Gene Set Enrichment Analysis (GSEA)

GSEA [36], [37] was performed using default parameters. Analyzed gene sets were comprised of all the genes defining human HCC subclasses S1, S2, and S3 [35] for which mouse orthologs have been annotated.

### Immunohistochemistry

Formalin-fixed, paraffin-embedded liver samples were sectioned to a thickness of 4 µm and baked onto glass slides. Samples were de-paraffinized, rehydrated, and treated with citrate antigen unmasking solution (Vector Laboratories). Endogenous peroxidase activity was blocked by treatment with a 3% solution of hydrogen peroxide for fifteen minutes. The anti-rabbit ImmPRESS reagent kit (Vector Laboratories) was used for immunolabeling with primary antibodies anti-Fyb (Abgent #AJ1306a) and anti-Ier3 (Abgent #AP11790a). Both primary antibodies were diluted 1∶100 and incubated with samples for one hour at room temperature. The ImmPACT DAB kit (Vector Laboratories) was used for detection. Sections were counterstained with hematoxylin QS (Vector Laboratories) and mounted in Permount (Fisher Scientific) for light microscopy.

## Supporting Information

Figure S1 Heat map depicting global differential transcript expression in SB-induced HCCs and normal liver. Unsupervised clustering was performed based on genes with normalized expression values varying among samples by at least 2.5 standard deviations. For genes with more than one associated transcript, the NCBI RefSeq accession number is indicated. *Rtl1* is indicated with a red arrow.
(TIF)

Figure S2 Heat map depicting global differential miRNA expression in SB-induced HCCs and normal liver. Unsupervised clustering was performed based on miRNAs with normalized expression values varying among samples by at least 1.5 standard deviations. *Dlk1-Dio3* domain miRNAs are listed in red.
(TIF)

Figure S3 Transposon integrations are preferentially detected upstream of *Rtl1* in SB-induced HCCs. (A) For this analysis, the *Dlk1-Dio3* domain was divided into eleven distinct regions defined by constituent genes and their promoter regions. (B) Quantification of *Dlk1-Dio3* domain transposon integrations in the livers of SB mice. A comprehensive analysis of all transposon insertions within chromosome 12 detected in six normal livers and thirty-four HCCs from SB mice. Insertions are grouped into three distinct categories based on whether they were detected in normal liver or tumor tissue and whether they were identified as clonal or subclonal (none of the sites identified in normal tissue were identified as clonal). The bar graph shows percentages of all chromosome 12 insertions that fall within the intervals defined in panel A. The actual values used to generate the graph are shown in the adjacent table. (C) Analysis of this larger set of tumors confirms the selection and orientation bias for transposon integrations upstream of *Rtl1*. Filled arrowheads represent transposons with the same transcriptional as *Rtl1* and unfilled arrowheads represent transposons with the opposite orientation. Each arrowhead represents a clonal insertion detected in a separate tumor sample (IGR = intergenic region).
(TIF)

Figure S4 Validation of transgene expression in tumors induced by hydrodynamic injection. (A) Confirmation of Trp53 knockdown in tumors from mice injected with p53 hairpin construct. Western blotting was used to detect the presence of the pT2/shp53 construct and its knockdown efficiency in tumors. Detection of GFP indicates presence of the construct, which also contains a GFP expression cassette. To assess the degree of knockdown, Trp53 signal for each sample was normalized to beta-tubulin signal from the same sample. For each tumor, this ratio was normalized to the ratio obtained for a tumor developed following hydrodynamic injection without the p53 hairpin construct (*Rtl1* only tumor). These normalized values are plotted in the graph below. (B) PCR on cDNA (RT +) confirmed expression of *Rtl1* and *Fah* in eleven of fourteen tumors. Control reactions performed without reverse transcriptase (RT −) are also shown. Amplification of *Tbp* was included as a control for cDNA quality.
(TIF)

Figure S5 Gel images of the RT-PCR used to generate Figure 5A. Expression of *RTL1* in a set of human tumors (T) and matched benign tissue (B) were analyzed by RT-PCR (RT +). Control reactions performed without reverse transcriptase (RT −) are also shown. Amplification of *TBP* was included to allow normalization for template amounts.
(TIF)

Figure S6 Validation of S1 subclass protein expression in SB-induced HCC. Immunohistochemistry was used to confirm altered expression of two proteins from the human HCC subclass S1 gene set. (A–B) Staining for FYN binding protein (Fyb) was performed on normal liver (A) and HCC tissue (B) from a mouse with SB-induced HCC. Though detected in both tissues, the staining pattern in normal liver is more diffuse. Regions of higher staining density are detected specifically in the tumor. (C–D) Staining for Immediate early response 3 (Ier3) was performed on the same tissue samples shown in panels A–B. No significant expression was detected in normal liver, while several regions of high density staining were detected in the tumor. (E) Section of the same tumor used in panels B and D for which the primary antibody was omitted. Scale bars = 100 µm.
(TIF)

Table S1 Global transcriptome sequencing data.
(XLSX)

Table S2 Global miRNA sequencing data.
(XLSX)

## References

[1] Ferlay J, Shin HR, Bray F, Forman D, Mathers C, et al (2010) Estimates of worldwide burden of cancer in 2008: GLOBOCAN 2008. Int J Cancer 127: 2893–2917. 21351269 10.1002/ijc.25516

[2] Altekruse SF, McGlynn KA, Reichman ME (2009) Hepatocellular carcinoma incidence, mortality, and survival trends in the United States from 1975 to 2005. J Clin Oncol 27: 1485–1491. 19224838 10.1200/JCO.2008.20.7753 PMC2668555

[3] Llovet JM, Ricci S, Mazzaferro V, Hilgard P, Gane E, et al (2008) Sorafenib in advanced hepatocellular carcinoma. N Engl J Med 359: 378–390. 18650514 10.1056/NEJMoa0708857

[4] Howell VM (2012) Sleeping beauty–a mouse model for all cancers? Cancer Lett 317: 1–8. 22079740 10.1016/j.canlet.2011.11.006

[5] Dupuy AJ, Rogers LM, Kim J, Nannapaneni K, Starr TK, et al (2009) A modified sleeping beauty transposon system that can be used to model a wide variety of human cancers in mice. Cancer Res 69: 8150–8156. 19808965 10.1158/0008-5472.CAN-09-1135 PMC3700628

[6] da Rocha ST, Edwards CA, Ito M, Ogata T, Ferguson-Smith AC (2008) Genomic imprinting at the mammalian Dlk1-Dio3 domain. Trends Genet 24: 306–316. 18471925 10.1016/j.tig.2008.03.011

[7] Georgiades P, Watkins M, Surani MA, Ferguson-Smith AC (2000) Parental origin-specific developmental defects in mice with uniparental disomy for chromosome 12. Development 127: 4719–4728. 11023874 10.1242/dev.127.21.4719

[8] Ogata T, Kagami M, Ferguson-Smith AC (2008) Molecular mechanisms regulating phenotypic outcome in paternal and maternal uniparental disomy for chromosome 14. Epigenetics 3: 181–187. 18698157 10.4161/epi.3.4.6550

[9] Donsante A, Miller DG, Li Y, Vogler C, Brunt EM, et al (2007) AAV vector integration sites in mouse hepatocellular carcinoma. Science 317: 477. 17656716 10.1126/science.1142658

[10] Wang PR, Xu M, Toffanin S, Li Y, Llovet JM, et al (2012) Induction of hepatocellular carcinoma by in vivo gene targeting. Proc Natl Acad Sci U S A 109: 11264–11269. 22733778 10.1073/pnas.1117032109 PMC3396480

[11] Braconi C, Kogure T, Valeri N, Huang N, Nuovo G, et al (2011) microRNA-29 can regulate expression of the long non-coding RNA gene MEG3 in hepatocellular cancer. Oncogene 30: 4750–4756. 21625215 10.1038/onc.2011.193 PMC4292930

[12] Huang J, Zhang X, Zhang M, Zhu JD, Zhang YL, et al (2007) Up-regulation of DLK1 as an imprinted gene could contribute to human hepatocellular carcinoma. Carcinogenesis 28: 1094–1103. 17114643 10.1093/carcin/bgl215

[13] Luk JM, Burchard J, Zhang C, Liu AM, Wong KF, et al (2011) DLK1-DIO3 genomic imprinted microRNA cluster at 14q32.2 defines a stemlike subtype of hepatocellular carcinoma associated with poor survival. J Biol Chem 286: 30706–30713. 21737452 10.1074/jbc.M111.229831 PMC3162431

[14] Yu F, Hao X, Zhao H, Ge C, Yao M, et al (2010) Delta-like 1 contributes to cell growth by increasing the interferon-inducible protein 16 expression in hepatocellular carcinoma. Liver Int 30: 703–714. 20214740 10.1111/j.1478-3231.2010.02214.x

[15] Ranzani M, Cesana D, Bartholomae CC, Sanvito F, Pala M, et al (2013) Lentiviral vector-based insertional mutagenesis identifies genes associated with liver cancer. Nat Methods 10: 155–161. 23314173 10.1038/nmeth.2331 PMC3589714

[16] Youngson NA, Kocialkowski S, Peel N, Ferguson-Smith AC (2005) A small family of sushi-class retrotransposon-derived genes in mammals and their relation to genomic imprinting. J Mol Evol 61: 481–490. 16155747 10.1007/s00239-004-0332-0

[17] Edwards CA, Mungall AJ, Matthews L, Ryder E, Gray DJ, et al (2008) The evolution of the DLK1-DIO3 imprinted domain in mammals. PLoS Biol 6: e135 doi:10.1371/journal.pbio.0060135. 18532878 10.1371/journal.pbio.0060135 PMC2408620

[18] Steshina EY, Carr MS, Glick EA, Yevtodiyenko A, Appelbe OK, et al (2006) Loss of imprinting at the Dlk1-Gtl2 locus caused by insertional mutagenesis in the Gtl2 5′ region. BMC Genet 7: 44. 17014736 10.1186/1471-2156-7-44 PMC1609179

[19] Seitz H, Youngson N, Lin SP, Dalbert S, Paulsen M, et al (2003) Imprinted microRNA genes transcribed antisense to a reciprocally imprinted retrotransposon-like gene. Nat Genet 34: 261–262. 12796779 10.1038/ng1171

[20] Brett BT, Berquam-Vrieze KE, Nannapaneni K, Huang J, Scheetz TE, et al (2011) Novel molecular and computational methods improve the accuracy of insertion site analysis in Sleeping Beauty-induced tumors. PLoS ONE 6: e24668 doi:10.1371/journal.pone.0024668. 21931803 10.1371/journal.pone.0024668 PMC3172244

[21] Li X, Ewis H, Hice RH, Malani N, Parker N, et al (2013) A resurrected mammalian hAT transposable element and a closely related insect element are highly active in human cell culture. Proc Natl Acad Sci U S A 110: E478–487. 23091042 10.1073/pnas.1121543109 PMC3568352

[22] Woodard LE, Li X, Malani N, Kaja A, Hice RH, et al (2012) Comparative analysis of the recently discovered hAT transposon TcBuster in human cells. PLoS ONE 7: e42666 doi:10.1371/journal.pone.0042666. 23166581 10.1371/journal.pone.0042666 PMC3499496

[23] O'Donnell KA, Keng VW, York B, Reineke EL, Seo D, et al (2012) A Sleeping Beauty mutagenesis screen reveals a tumor suppressor role for Ncoa2/Src-2 in liver cancer. Proc Natl Acad Sci U S A 109: E1377–1386. 22556267 10.1073/pnas.1115433109 PMC3361419

[24] Keng VW, Villanueva A, Chiang DY, Dupuy AJ, Ryan BJ, et al (2009) A conditional transposon-based insertional mutagenesis screen for genes associated with mouse hepatocellular carcinoma. Nat Biotechnol 27: 264–274. 19234449 10.1038/nbt.1526 PMC2712727

[25] Harri L, Philippe C, Federico B, Arne M, Valerie D, et al (2012) Identification of Dlk1-Dio3 imprinted gene cluster non-coding RNAs as novel candidate biomarkers for liver tumor promotion. Toxicol Sci 131: 375–86. 23091169 10.1093/toxsci/kfs303

[26] Seitz HK, Stickel F (2006) Risk factors and mechanisms of hepatocarcinogenesis with special emphasis on alcohol and oxidative stress. Biol Chem 387: 349–360. 16606331 10.1515/BC.2006.047

[27] Zhang DY, Friedman SL (2012) Fibrosis-dependent mechanisms of hepatocarcinogenesis. Hepatology 56: 769–775. 22378017 10.1002/hep.25670 PMC4087159

[28] Bell JB, Podetz-Pedersen KM, Aronovich EL, Belur LR, McIvor RS, et al (2007) Preferential delivery of the Sleeping Beauty transposon system to livers of mice by hydrodynamic injection. Nat Protoc 2: 3153–3165. 18079715 10.1038/nprot.2007.471 PMC2548418

[29] Wangensteen KJ, Wilber A, Keng VW, He Z, Matise I, et al (2008) A facile method for somatic, lifelong manipulation of multiple genes in the mouse liver. Hepatology 47: 1714–1724. 18435462 10.1002/hep.22195 PMC5808937

[30] Grompe M, Overturf K, al-Dhalimy M, Finegold M (1998) Therapeutic trials in the murine model of hereditary tyrosinaemia type I: a progress report. J Inherit Metab Dis 21: 518–531. 9728332 10.1023/a:1005462804271

[31] Keng VW, Tschida BR, Bell JB, Largaespada DA (2011) Modeling hepatitis B virus X-induced hepatocellular carcinoma in mice with the Sleeping Beauty transposon system. Hepatology 53: 781–790. 21374658 10.1002/hep.24091 PMC3079950

[32] Shuldiner AR, Nirula A, Roth J (1990) RNA template-specific polymerase chain reaction (RS-PCR): a novel strategy to reduce dramatically false positives. Gene 91: 139–142. 1698167 10.1016/0378-1119(90)90176-r

[33] Feinberg AP, Tycko B (2004) The history of cancer epigenetics. Nat Rev Cancer 4: 143–153. 14732866 10.1038/nrc1279

[34] Holm TM, Jackson-Grusby L, Brambrink T, Yamada Y, Rideout WM 3rd, et al (2005) Global loss of imprinting leads to widespread tumorigenesis in adult mice. Cancer Cell 8: 275–285. 16226703 10.1016/j.ccr.2005.09.007

[35] Hoshida Y, Nijman SM, Kobayashi M, Chan JA, Brunet JP, et al (2009) Integrative transcriptome analysis reveals common molecular subclasses of human hepatocellular carcinoma. Cancer Res 69: 7385–7392. 19723656 10.1158/0008-5472.CAN-09-1089 PMC3549578

[36] Mootha VK, Lindgren CM, Eriksson KF, Subramanian A, Sihag S, et al (2003) PGC-1alpha-responsive genes involved in oxidative phosphorylation are coordinately downregulated in human diabetes. Nat Genet 34: 267–273. 12808457 10.1038/ng1180

[37] Subramanian A, Tamayo P, Mootha VK, Mukherjee S, Ebert BL, et al (2005) Gene set enrichment analysis: a knowledge-based approach for interpreting genome-wide expression profiles. Proc Natl Acad Sci U S A 102: 15545–15550. 16199517 10.1073/pnas.0506580102 PMC1239896

[38] Sekita Y, Wagatsuma H, Nakamura K, Ono R, Kagami M, et al (2008) Role of retrotransposon-derived imprinted gene, Rtl1, in the feto-maternal interface of mouse placenta. Nat Genet 40: 243–248. 18176565 10.1038/ng.2007.51

[39] Marciniszyn J Jr, Hartsuck JA, Tang J (1976) Mode of inhibition of acid proteases by pepstatin. J Biol Chem 251: 7088–7094. 993206

[40] Wood JM, Maibaum J, Rahuel J, Grutter MG, Cohen NC, et al (2003) Structure-based design of aliskiren, a novel orally effective renin inhibitor. Biochem Biophys Res Commun 308: 698–705. 12927775 10.1016/s0006-291x(03)01451-7

[41] Robinson BS, Riccardi KA, Gong YF, Guo Q, Stock DA, et al (2000) BMS-232632, a highly potent human immunodeficiency virus protease inhibitor that can be used in combination with other available antiretroviral agents. Antimicrob Agents Chemother 44: 2093–2099. 10898681 10.1128/aac.44.8.2093-2099.2000 PMC90019

[42] Mortazavi A, Williams BA, McCue K, Schaeffer L, Wold B (2008) Mapping and quantifying mammalian transcriptomes by RNA-Seq. Nat Methods 5: 621–628. 18516045 10.1038/nmeth.1226 PMC13303166

[43] de Hoon MJ, Imoto S, Nolan J, Miyano S (2004) Open source clustering software. Bioinformatics 20: 1453–1454. 14871861 10.1093/bioinformatics/bth078

[44] Saldanha AJ (2004) Java Treeview–extensible visualization of microarray data. Bioinformatics 20: 3246–3248. 15180930 10.1093/bioinformatics/bth349

[45] Dickins RA, Hemann MT, Zilfou JT, Simpson DR, Ibarra I, et al (2005) Probing tumor phenotypes using stable and regulated synthetic microRNA precursors. Nat Genet 37: 1289–1295. 16200064 10.1038/ng1651

