# Supplementary figures and images for: Identification of Rtl1, a Retrotransposon-Derived Imprinted Gene, as a Novel Driver of Hepatocarcinogenesis
Source: PLoS Genet. 2013 Apr 4;9(4):e1003441. doi: 10.1371/journal.pgen.1003441 (PMC3616914; doi:10.1371/journal.pgen.1003441)

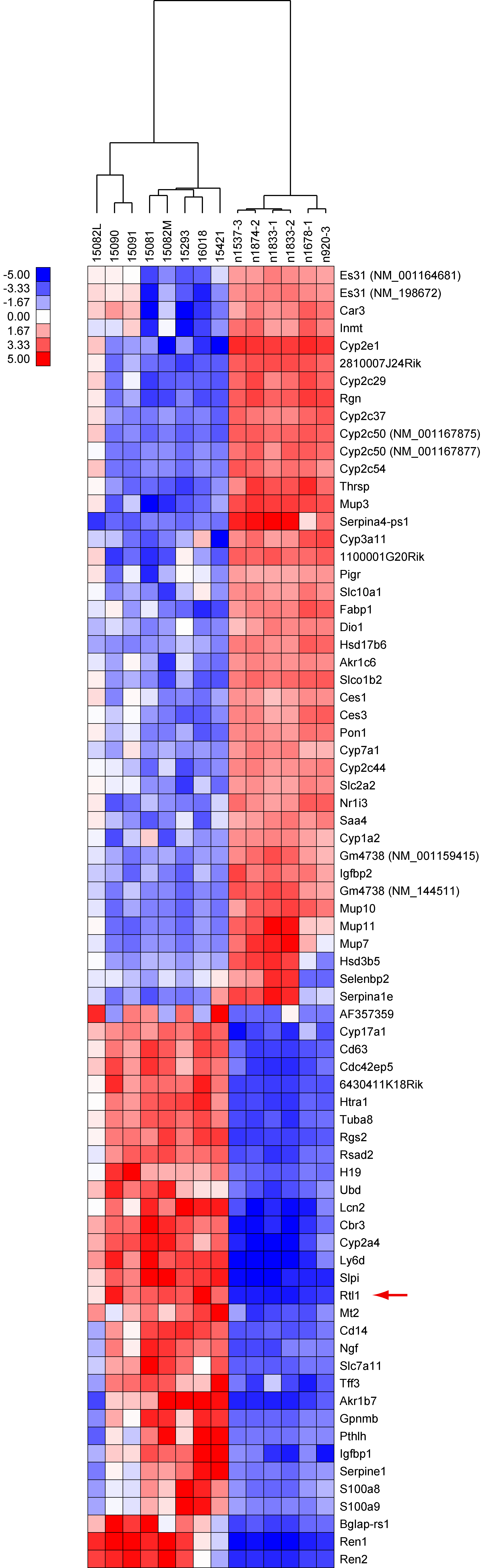

Supplement: Figure S1 — Heat map depicting global differential transcript expression in SB-induced HCCs and normal liver. Unsupervised clustering was performed based on genes with normalized expression values varying among samples by at least 2.5 standard deviations. For genes with more than one associated transcript, the NCBI RefSeq accession number is indicated. Rtl1 is indicated with a red arrow. (TIF) [file pgen.1003441.s001.tif]

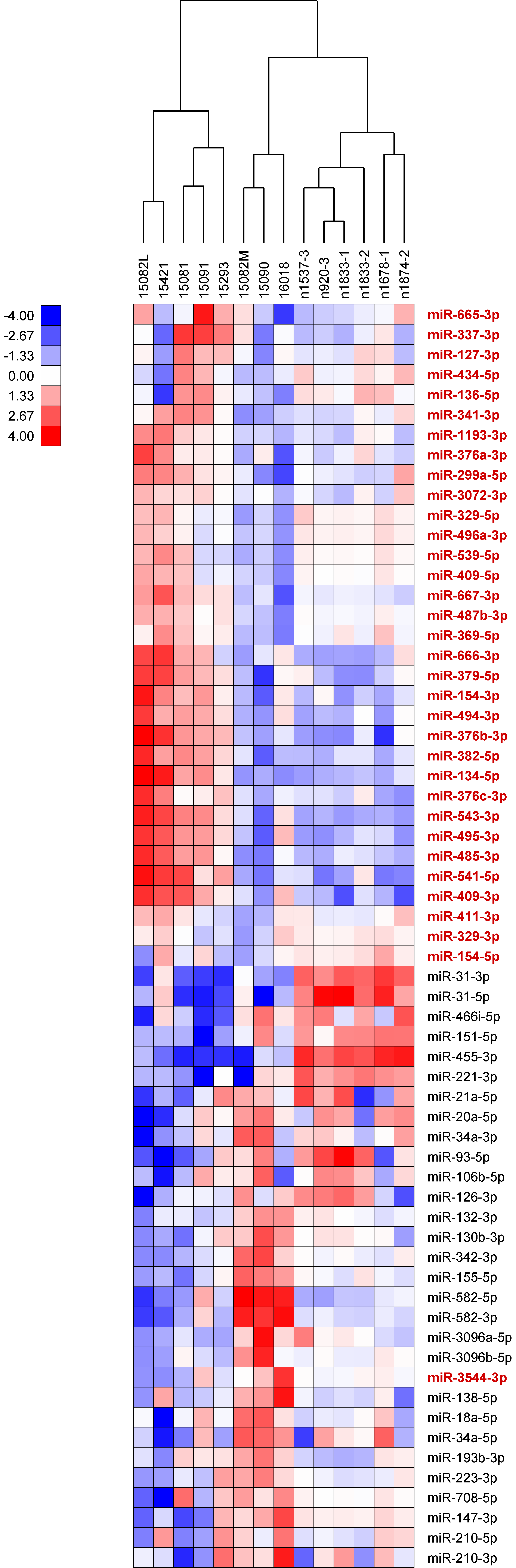

Supplement: Figure S2 — Heat map depicting global differential miRNA expression in SB-induced HCCs and normal liver. Unsupervised clustering was performed based on miRNAs with normalized expression values varying among samples by at least 1.5 standard deviations. Dlk1-Dio3 domain miRNAs are listed in red. (TIF) [file pgen.1003441.s002.tif]

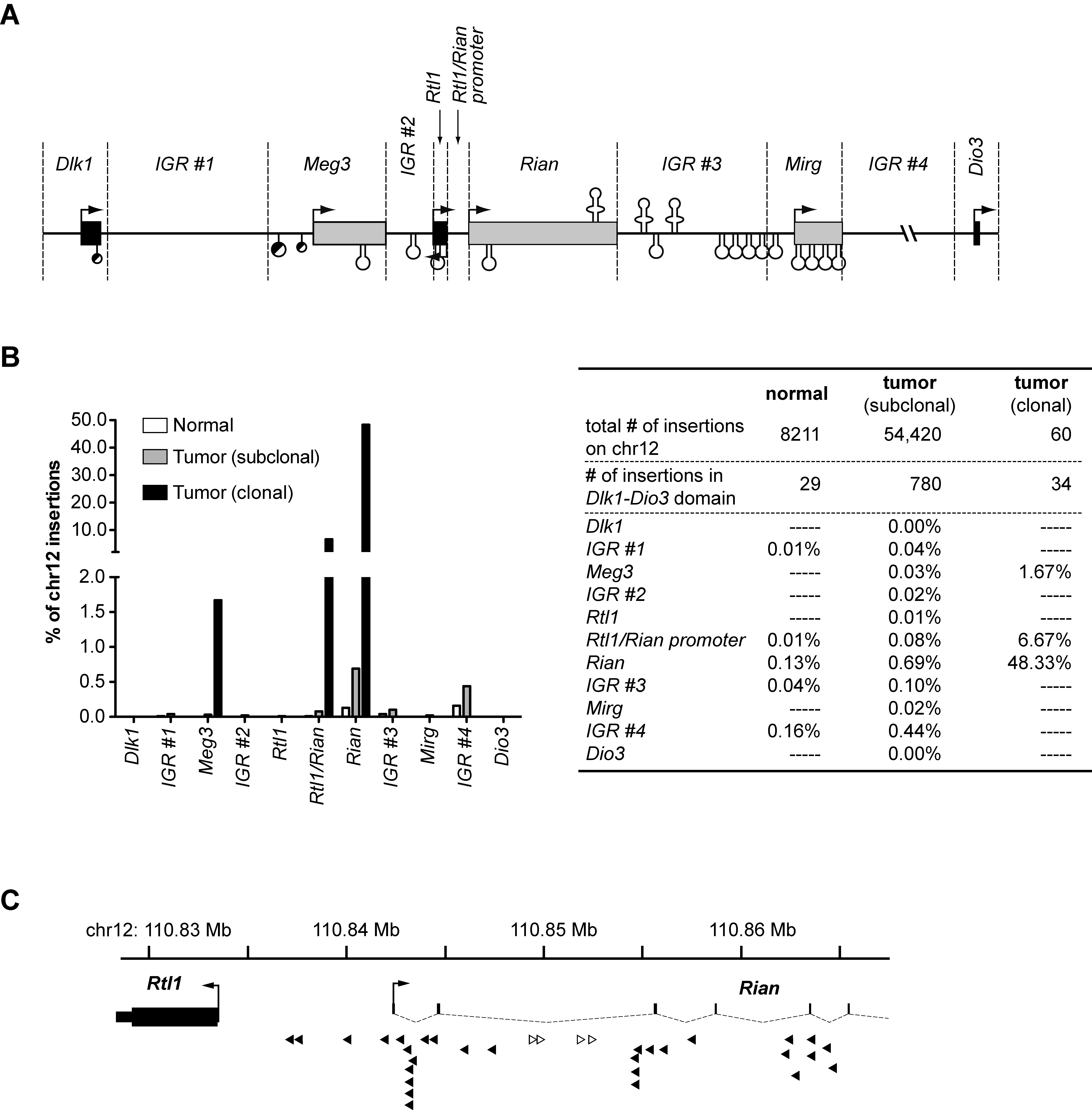

Supplement: Figure S3 — Transposon integrations are preferentially detected upstream of Rtl1 in SB-induced HCCs. (A) For this analysis, the Dlk1-Dio3 domain was divided into eleven distinct regions defined by constituent genes and their promoter regions. (B) Quantification of Dlk1-Dio3 domain transposon integrations in the livers of SB mice. A comprehensive analysis of all transposon insertions within chromosome 12 detected in six normal livers and thirty-four HCCs from SB mice. Insertions are grouped into three distinct categories based on whether they were detected in normal liver or tumor tissue and whether they were identified as clonal or subclonal (none of the sites identified in normal tissue were identified as clonal). The bar graph shows percentages of all chromosome 12 insertions that fall within the intervals defined in panel A. The actual values used to generate the graph are shown in the adjacent table. (C) Analysis of this larger set of tumors confirms the selection and orientation bias for transposon integrations upstream of Rtl1. Filled arrowheads represent transposons with the same transcriptional as Rtl1 and unfilled arrowheads represent transposons with the opposite orientation. Each arrowhead represents a clonal insertion detected in a separate tumor sample (IGR = intergenic region). (TIF) [file pgen.1003441.s003.tif]

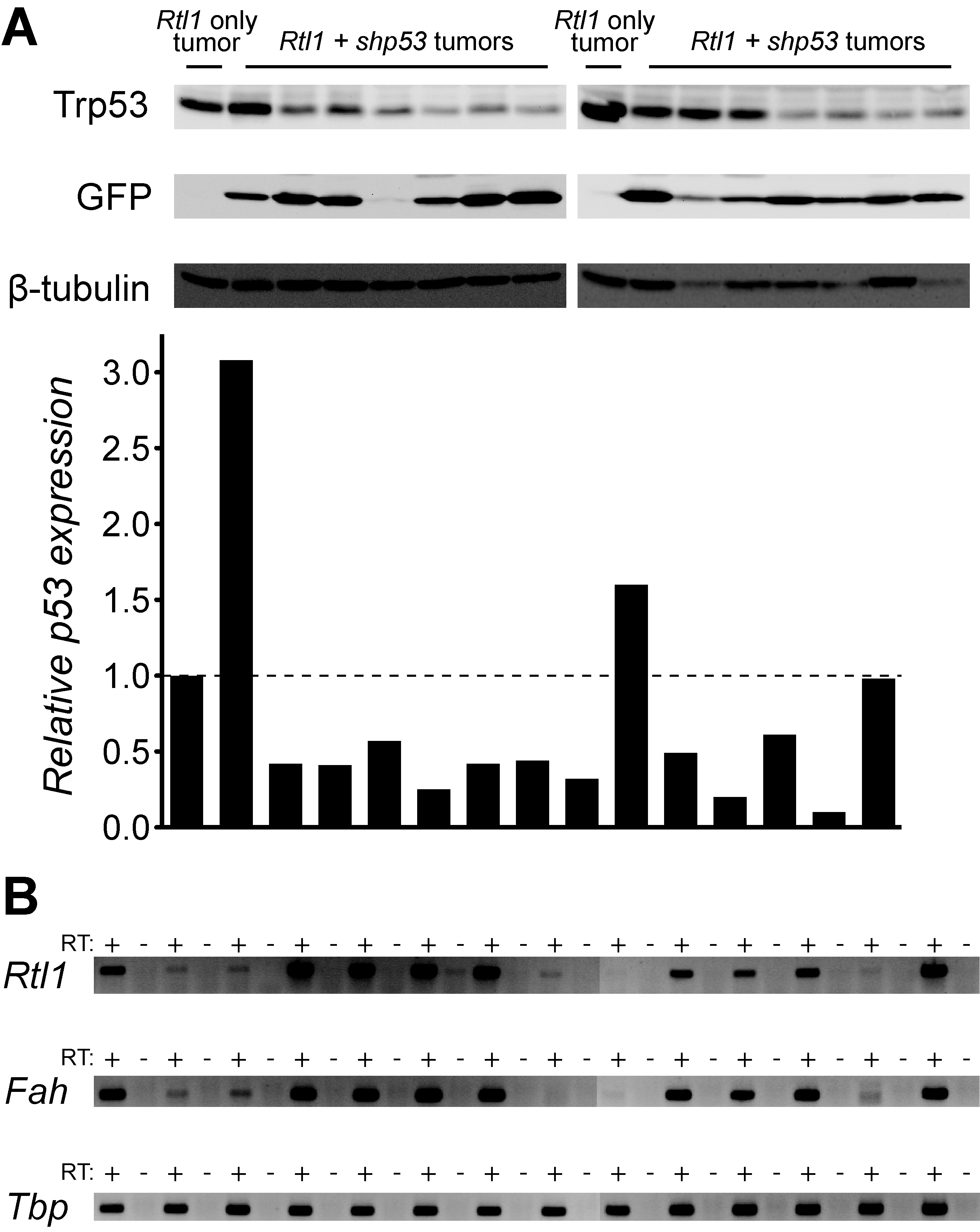

Supplement: Figure S4 — Validation of transgene expression in tumors induced by hydrodynamic injection. (A) Confirmation of Trp53 knockdown in tumors from mice injected with p53 hairpin construct. Western blotting was used to detect the presence of the pT2/shp53 construct and its knockdown efficiency in tumors. Detection of GFP indicates presence of the construct, which also contains a GFP expression cassette. To assess the degree of knockdown, Trp53 signal for each sample was normalized to beta-tubulin signal from the same sample. For each tumor, this ratio was normalized to the ratio obtained for a tumor developed following hydrodynamic injection without the p53 hairpin construct (Rtl1 only tumor). These normalized values are plotted in the graph below. (B) PCR on cDNA (RT +) confirmed expression of Rtl1 and Fah in eleven of fourteen tumors. Control reactions performed without reverse transcriptase (RT −) are also shown. Amplification of Tbp was included as a control for cDNA quality. (TIF) [file pgen.1003441.s004.tif]

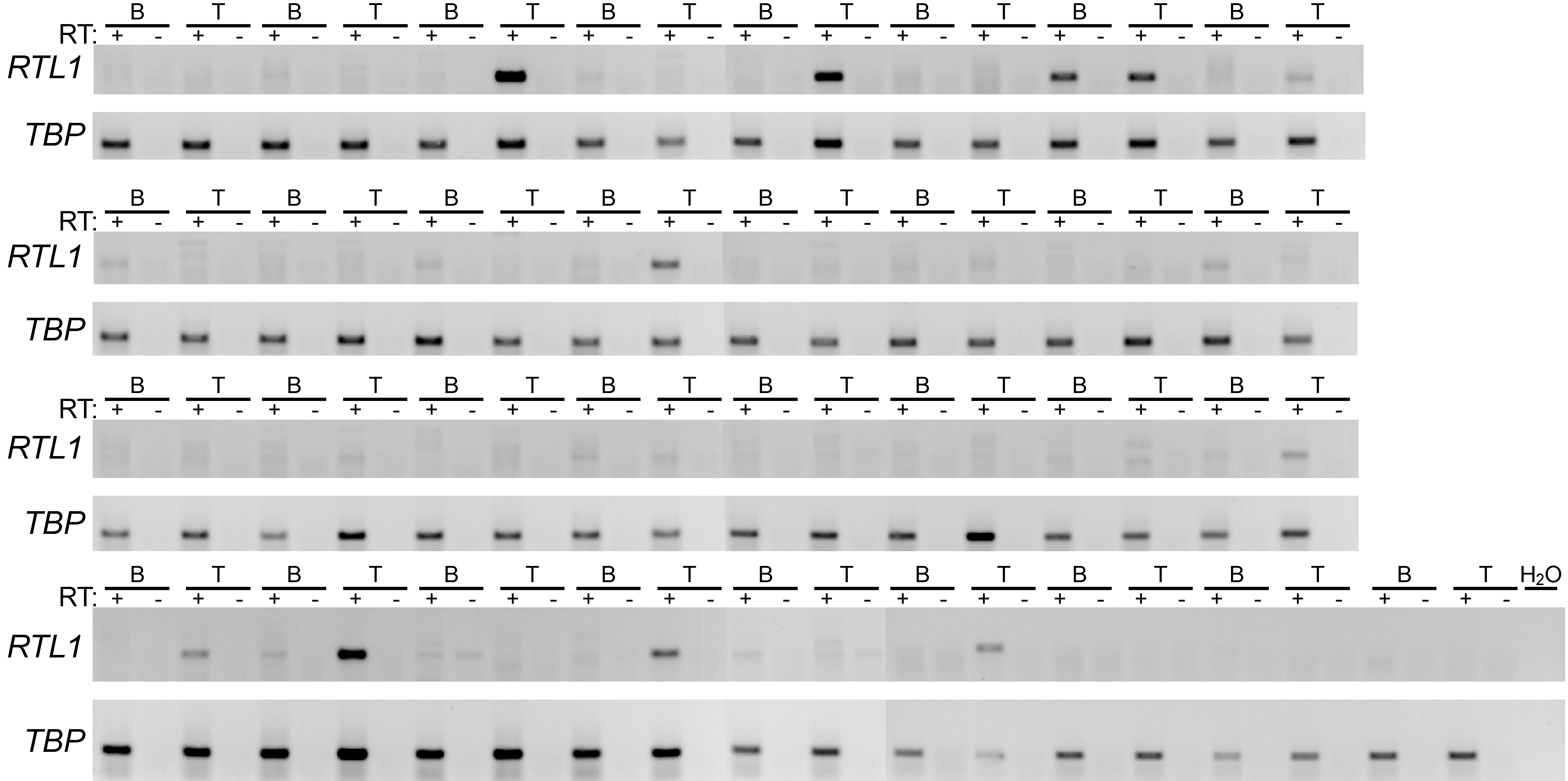

Supplement: Figure S5 — Gel images of the RT-PCR used to generate Figure 5A. Expression of RTL1 in a set of human tumors (T) and matched benign tissue (B) were analyzed by RT-PCR (RT +). Control reactions performed without reverse transcriptase (RT −) are also shown. Amplification of TBP was included to allow normalization for template amounts. (TIF) [file pgen.1003441.s005.tif]

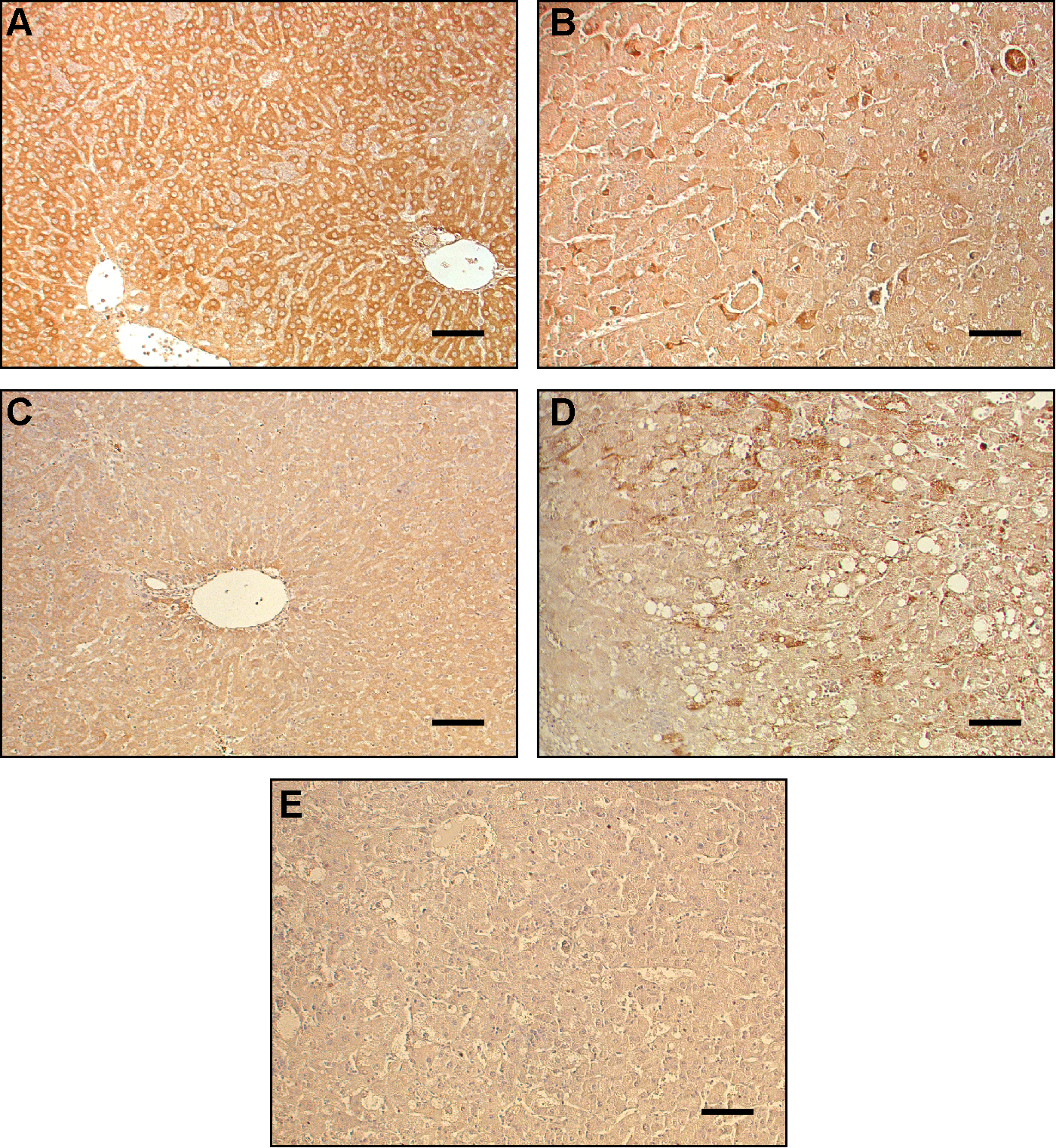

Supplement: Figure S6 — Validation of S1 subclass protein expression in SB-induced HCC. Immunohistochemistry was used to confirm altered expression of two proteins from the human HCC subclass S1 gene set. (A–B) Staining for FYN binding protein (Fyb) was performed on normal liver (A) and HCC tissue (B) from a mouse with SB-induced HCC. Though detected in both tissues, the staining pattern in normal liver is more diffuse. Regions of higher staining density are detected specifically in the tumor. (C–D) Staining for Immediate early response 3 (Ier3) was performed on the same tissue samples shown in panels A–B. No significant expression was detected in normal liver, while several regions of high density staining were detected in the tumor. (E) Section of the same tumor used in panels B and D for which the primary antibody was omitted. Scale bars = 100 µm. (TIF) [file pgen.1003441.s006.tif]
